# Sustained Release of Decoy Wnt Receptor (sLRP6E1E2)-Expressing Adenovirus Using Gel-Encapsulation for Scar Remodeling in Pig Model

**DOI:** 10.3390/ijms21062242

**Published:** 2020-03-24

**Authors:** Chae-Eun Yang, Sewoon Choi, Ju Hee Lee, Eun Hye Kang, Hyo Min Ahn, Tai Suk Roh, Chae-Ok Yun, Won Jai Lee

**Affiliations:** 1 Department of Plastic & Reconstructive Surgery, Wonju College of Medicine, Yonsei University, Wonju 26426, Korea; cheniya@yuhs.ac; 2 Institute for Human Tissue Restoration, Department of Plastic & Reconstructive Surgery, Yonsei University College of Medicine, Seoul 03722, Korea; 3 Department of Dermatology and Cutaneous Biology Research Institute, Yonsei University College of Medicine, Seoul 03722, Korea; 4 Department of Bioengineering, College of Engineering, Hanyang University, Seoul 04763, Korea; 5 Institute of Nano Science and Technology (INST), Hanyang University, Seoul 04763, Korea

**Keywords:** gene therapy, adenovirus, scar, alginate gel, decoy Wnt receptor, pig model

## Abstract

An adenoviral vector (Ad) expressing a Wnt decoy receptor (sLRP6E1E2) is known to induce an anti-fibrotic effect by inhibiting Wnt signaling. We evaluated its effects in vivo using pig models and attempted to introduce an alginate gel-matrix system to prolong the effect of the Ad. Transduction efficiency as to the biological activity of Ad in different forms was evaluated. Then, 50 days after the formation of full-thickness skin defects on the backs of Yorkshire pigs, scars were treated with each form of Ad. Therapeutic efficacy and various factors influencing scar formation and collagen rearrangement were analyzed. Inflammatory cell infiltration within the scar tissues was also evaluated. Decoy Wnt receptor (sLRP6E1E2)-expressing adenovirus treatment improved scar quality in a pig model. Loading this construct in alginate gel allows sustained virus release into local tissues and prolongs Ad activity, thus maintaining its therapeutic effect longer in vivo.

## 1. Introduction

Some wounds undergo an abnormal healing process and leave disfiguring scars. Hypertrophic scars and keloids are representative examples of healed skin that are exceptionally red, thick, protruded, and tight. Various mechanisms have been proposed to explain keloid pathogenesis, but the treatment of keloids is extremely difficult as keloids frequently form at the site of injury, recur after excision, and always overgrow beyond the boundaries of the original wound. This abnormal scarring is usually the result of unbalanced collagen production and degradation. Chronic, persistent, and prolonged proliferation of fibroblast leads to excessive synthesis and deposition of extracellular matrix (ECM) components, especially collagen. Although patients usually suffer from aesthetic disturbances and functional problems, these scars are difficult to treat due to their complex pathophysiology and unknown polygenetic precipitating factors [1,2,3]. The response to treatment among patients is highly variable, and currently available therapies often achieve only temporary improvement [1,4,5].

Transforming growth factor-β (TGF-β) is a key regulator of fibroblast activation that drives the excessive synthesis of ECM in fibrotic diseases. Recently, signaling cross-talk between the Wnt/β-catenin, TGF-β1, and Smads was demonstrated, where Wnt/β-catenin signaling activates TGF-β19, 15 and TGF-β1 promotes Wnt/β-catenin signaling. Therefore, inhibition of the canonical Wnt pathway can be an effective approach to target TGF-β1 signaling, which can both synergistically downregulate fibrogenesis. Therefore, on our previous research, we have demonstrated that soluble Wnt decoy receptor of LRP6 (sLRP6E1E2)-expressing Ad (dE1-k35/sLRP6E1E2), which expresses LRP6’ E1 and E2 domains, prevents Wnt-mediated stabilization of cytoplasmic β-catenin, and decreases Wnt/β-catenin signaling, and this construct can degrade extracellular matrix in HDFs, KFs, and primary keloid spheroids, and thus it may be beneficial for the treatment of keloids [6].

Although adenoviral vectors are popular for gene therapy due to their high gene-transfer efficiency, they can deteriorate rapidly within a few weeks due to a short half-life, enzymatic inactivation, and transient effects. This may require repeated administration of an adenovirus to generate the desired anti-fibrotic effect, and regional administration of a high concentration of Ad can impair potential therapeutic effect and decrease safety. To deal with these problems, we loaded the virus in a sodium alginate-based hydrogel, which is a natural polymer frequently used in biomedical applications [7,8,9]. Alginate gel can be used as a depot system for the sustained release of Ad and maintaining Ad viral activity without affecting its biological activity [7,8]. Also, in previous research, we examined that the use of alginate gel could increase the therapeutic efficacy of adenovirus in a pig scar model [10].

In this study, we investigate the anti-fibrotic and scar remodeling effect of decoy Wnt receptor (sLRP6E1E2)-expressing adenovirus in vivo using a pig model. Additionally, we also demonstrate the use of an alginate gel-matrix system as a delivery vehicle to entrap sLRP6E1E2-expressing Ad for sustained release to promote scar tissue remodeling in a pig model.

## 2. Results

### 2.1. Ad Is Continuously Released from the Alginate Gel, and Gel Encapsulation Prolongs Ad Biological Activity

**Figure 1:**
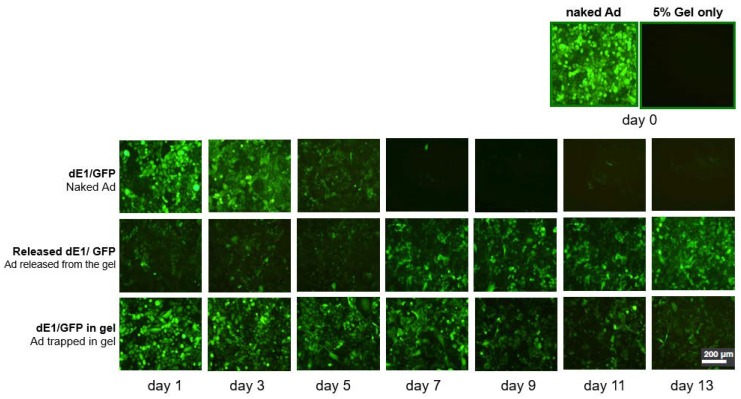
Comparisons of GFP (Green Fluorescent Protein) expression level by fluorescent microscopies showing SK-Hep1 cells incubated with adenoviral vector (Ad) in various forms. Ad (1 × 10^10^ viral particle of dE1/GFP) was encapsulated in 5% alginate gel and incubated with Dulbecco’s modified Eagle’s medium at 37 °C for 1, 3, 5, 7, 9, 11, and 13 days. SK-Hep1 cells transduced with dE1/GFP (naked Ad, top row), released Ad from 5% gel (middle row), and dissolved Ad-gel (lower row). GFP expression from naked Ad is strong at the beginning and dims after day 7, whereas from cells transduced with gel-release Ad accumulated amounts of released Ad gradually increased on day 13 versus day 1. Additionally, GFP expression of Ad in dissolved gel was prolonged until day 13. These results demonstrates that Ad in alginate gel is released over time in a sustained manner.

Transduction efficiency was compared by fluorescent micrographs showing SK-Hep1 cells incubated with Ad in different forms (Figure 1). GFP (Green Fluorescent Protein) expression of naked Ad was noticeably decreased from the 3rd day of incubation and almost absent after the 7th day (Figure 1, upper row), whereas cells transduced with 5% gel-released Ad showed relatively strong and sustained GFP expression until the 13th day (Figure 1, middle row). Also, GFP expression of Ad in dissolved gel was prolonged and much higher than naked Ad (Figure 1, lower row). These results demonstrate that the alginate gel provides a biocompatible environment for Ad to maintain its viral activity and release Ad in a sustained manner.

### 2.2. sLRP6E1E2-Expressing Ad/Gel Decreases scar Size and Color in Pig Scar Tissue

**Figure 2:**
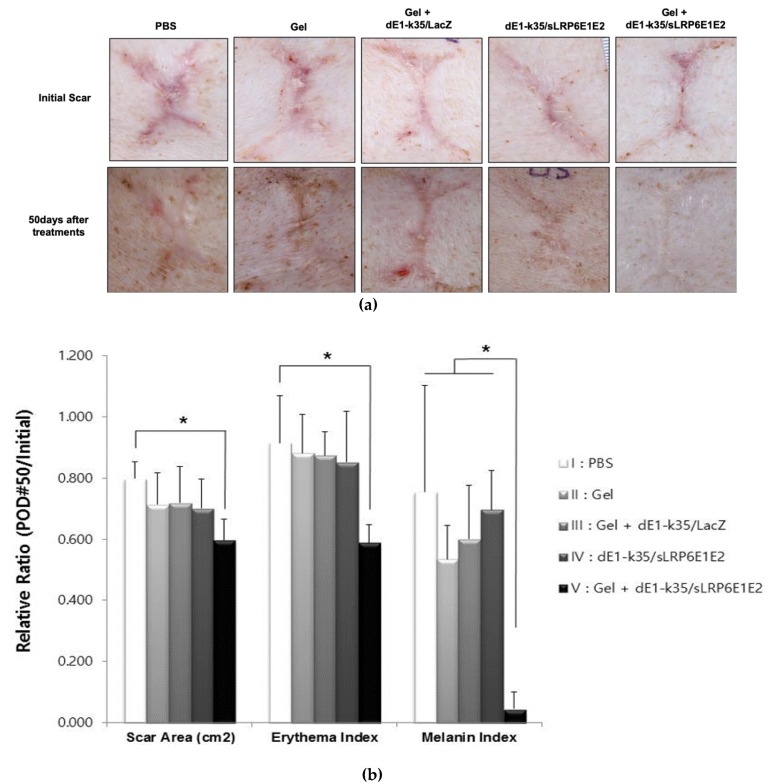
Photographic analysis and therapeutic evaluation of scar area and color indices with Image J software and spectrophotometry. (**a**) The photographs of scars before and 50 days after treatment in pigs of each group (Groups: I, PBS (control); II, alginate gel (gel); III, alginate gel encapsulating dE1-k35/LacZ (gel+dE1-k35/LacZ; capsulated control); IV, naked dE1-k35/sLRP6E1E2 (naked LRP6); and V, alginate gel encapsulating naked dE1-k35/sLRP6E1E2 (gel+dE1-k35/sLRP6E1E2, capsulated LRP6). (**b**) Therapeutic evaluation of the scars sLRP6E1E2-expressing Ad plays a prominent role in the reduction of scar surface area and color (mainly composed of erythema and melanin). Ad with alginate gel-matrix system showed more effective biologic activities than naked Ad.

The therapeutic effect of sLRP6E1E2-expressing Ad on scar tissue was evaluated with scar size and erythema and melanin indices. The values listed below correspond to the following treatment groups: I, PBS (control); II, alginate gel (gel); III, alginate gel encapsulating dE1-k35/LacZ (gel+dE1-k35/LacZ; capsulated control); IV, naked dE1-k35/sLRP6E1E2 (naked LRP6); and V, alginate gel encapsulating naked dE1-k35/sLRP6E1E2 (gel+dE1-k35/sLRP6E1E2, capsulated LRP6). Photographs of scars were taken before and after each treatment, and the scar area was measured with Image J software (Figure 2a). The sizes of initial scars were measured as 1.46 ± 0.88, 1.51 ± 0.79, 1.21 ± 0.15, 1.15 ± 0.18, and 1.42 ± 0.3 cm^2^ in each group, respectively, and there were no statistical differences. However, the sizes of the scars decreased to 79.8%, 71.3%, 71.9%, 70.1%, and 59.6% of initial scar, respectively, by the 50th day after treatments. This indicates that sLRP6E1E2 expression from Ad effectively reduced the size of the scar in Groups IV and V. Furthermore, in only Group V, gel encapsulating dE1-k35/sLRP6E1E2-treated scars showed a statistically significant reduction in scar area (*p* < 0.05; Figure 2b, left). 

The erythema indices of the initial scars were 2.9 ± 0.54, 3.12 ± 0.91, 2.77 ± 0.5, 2.32 ± 0.19, and 3.02 ± 0.48 in each group, respectively. Compared to baseline, the erythema index was decreased to 91.6%, 88.2%, 87.4%, 85.2%, and 58.9% of the initial value in each group, respectively, by the 50th day after treatments. The alginate gel encapsulating dE1-k35/sLRP6E1E2 treated scars (Group V) were significantly improved in terms of erythema compared to other groups (*p* < 0.05; Figure 2b, middle). Similarly, the melanin indices at 50 days were significantly reduced by 75.4%, 53.5%, 60.1%, 69.7%, and 4.6% in each group, respectively, showing that sLRP6E1E2 expression from gel-encapsulated Ad (Group V) reduces the melanin indices of scars (*p* < 0.05; Figure 2b, right). Taken together, these results indicate that sLRP6E1E2-expressing Ad plays a prominent role in improving scar size and color. Notably, Ad in an alginate gel-matrix system showed more effective biologic activities than naked Ad. 

### 2.3. sLRP6E1E2-Expressing Ad/Gel Remodels Scar by Inducing Collagen Rearrangement

**Figure 3:**
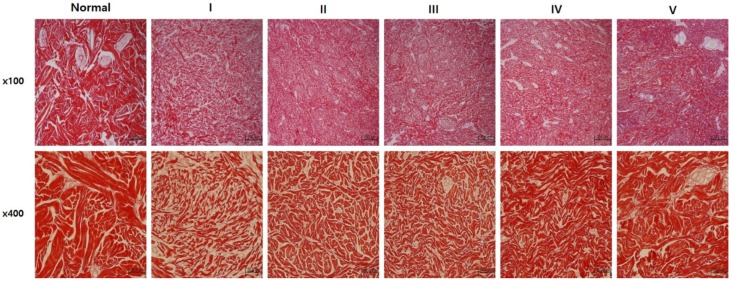
sLRP6E1E2-expressing Ad/gel induces collagen rearrangement. Picrosirius red staining in pig scar tissues. On the 50th day after treatment, premature collagen depositions were displaced by mature and well-arranged collagen bundles, and scar tissues in Groups IV and V had closely packed collagen fibers and formed complete bundles compared to other groups (Groups I to III), which is similar to normal dermal structure. Magnification × 100 (upper), × 400 (lower).

Tissues were stained with picrosirius red, which specifically binds collagen fibrils of various diameters. On the 50th day after treatment, scar tissues in Groups IV and V had closely packed collagen fibers and formed complete bundles compared to Groups I to III, which is similar to normal dermal structure (Figure 3). These data suggested that sLRP6E1E2 induces collagen rearrangement to resemble that of mature, bundle-shaped collagen fibers, and gel encapsulation may enhance its efficacy.

### 2.4. sLRP6E1E2-Expressing Ad/Gel Reduces Collagen I, Elastin, and Fibronectin Expression

**Figure 4:**
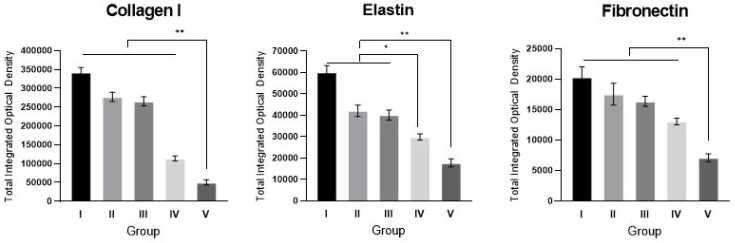
Representative sections of tissues from the center areas of the scar at 50th day after treatment were stained, and expression levels of type I collagen, elastin, and fibronectin were semi-quantitatively analyzed with MetaMorph^®^. Values are calculated as the means of the optical density of six different digital images. The expression levels of type I collagen, elastin, and fibronectin were significantly reduced in the alginate gel encapsulating dE1-k35/sLRP6E1E2-treated group (Group V) compared with other groups. Reduction in type I collagen was significant in Group IV compared to Groups I to III, but significantly higher (220%) than that of Group V (* *p* < 0.05, ** *p* < 0.01).

We examined the effects of sLRP6E1E2 overexpression on the major ECM components of scar tissues; type I collagen, elastin, and fibronectin. Immunohistochemical staining of scar sections revealed significant reductions in type I collagen and fibronectin in the alginate gel encapsulating dE1-k35/sLRP6E1E2-treated group (Group V) compared with other groups (** *p* < 0.01, Figure 4). Reductions of elastin in naked dE1-k35/sLRP6E1E2-treated scar tissues (Group IV) were significant compared to scars in Groups I, II, and III, but less than that of Group V. 

### 2.5. Alginate gel Encapsulating dE1-k35/sLRP6E1E2 Downregulates TGF-β1, Upregulates TGF-β3 mRNA Expression and Changes the Scar Remodeling Markers in Pig Scar Tissues 

**Figure 5:**
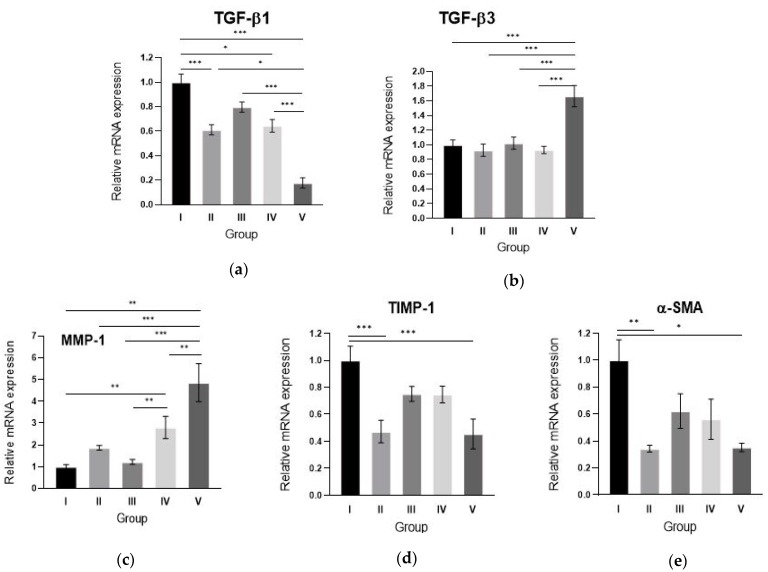
Alginate gel encapsulating dE1-k35/sLRP6E1E2 changes TGF-β mRNA expression and other scar remodeling markers in pig scar tissues. (**a**) TGF-β1 mRNA levels of Group V were 5-fold lower than that of Group I and 3-fold lower than that of Group IV scar tissues. (**b**) TGF-β3 mRNA levels of Group V were 1.6-fold higher than that of Group I scar tissues and 1.8-fold higher than that of Group IV scar tissues. (**c**) MMP-1 expression levels of Group V scar tissues were significantly increased by 4.9- and 1.7-fold versus Groups I and IV scar tissues, respectively (*** *p* < 0.001). In contrast, TIMP-1 (**d**) and α-SMA expression (**e**) levels of Group V scar tissues were significantly decreased by 2.2- and 2.8-fold versus Group I scar tissues, respectively (* *p* < 0.05, ** *p* < 0.01, *** *p* < 0.001).

To examine the mechanism by which sLRP6E1E2-expressing Ad suppresses expression of the major ECM components in pig scar tissues, the change of TGF-β1 and TGF-β3 mRNA expression levels were evaluated on various groups by qRT–PCR. The alginate gel encapsulating dE1-k35/sLRP6E1E2-treated scar tissues (group V) showed decreased TGF-β1 mRNA expression (Figure 5a) and increased TGF-β3 mRNA expression compared with other groups (Figure 5b). The TGF-β1 mRNA level of scar tissues from Group V were 5- and 3-fold lower than that of Groups I and IV, respectively (*p* < 0.001). In addition, TGF-β3 mRNA levels of Group V were 1.6-fold higher compared to Group I and 1.8-fold higher compared to Group IV (*p* < 0.001). These results suggest that the reduced expression of ECM components by sLRP6E1E2 overexpression is associated with decreased TGF-β1 expression and increased TGF-β3 expression. 

Scar remodeling-associated molecules such as MMP-1, TIMP-1, and α-SMA are important markers of the effects of TGF-β on wound repair. We performed a qRT–PCR analysis of MMP-1, TIMP-1, and α-SMA and showed differential expression of these matrix remodeling-associated molecules among experimental groups. MMP-1 expression levels of Group V scar tissues were significantly increased by 4.9- and 1.7-fold, respectively, versus those of Groups I and IV scar tissues (*p* < 0.001, Figure 5c). In contrast, TIMP-1 and α-SMA expression levels of Group V scar tissues were significantly decreased by 2.2- and 2.8-fold, respectively, compared to Groups I and II (*p* < 0.01, Figure 5d,e). However, there was no significant difference between Groups IV and V. These results suggest that sLRP6E1E2 upregulates MMP-1 and downregulates TIMP-1 and α-SMA, which are major players in collagen breakdown. 

### 2.6. Alginate Gel Encapsulating dE1-k35/sLRP6E1E2 Decreases Inflammatory Cell Counts and Mast Cell Counts in Pig Scar Tissues 

**Figure 6:**
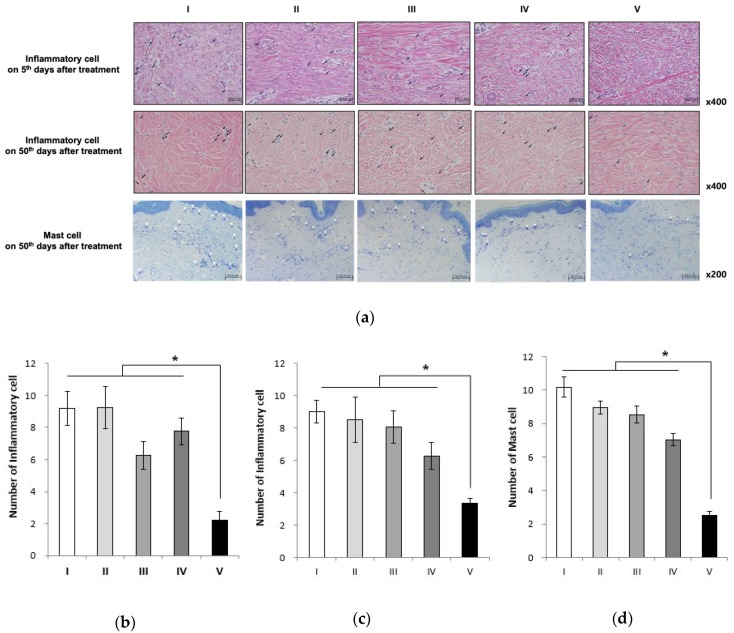
Inflammatory cell and mast cell count in pig scar tissue. (**a**) Inflammatory cell (black arrow) infiltration, as demonstrated by H&E staining (magnification, 400×) and mast cell (white arrowhead) count with toluidine blue staining in the scar area. The numbers of inflammatory cells on day 5 (**b**) and day 50 (**c**) were significantly lower in the alginate gel encapsulating dE1-k35/sLRP6E1E2-treated scar tissues (Group V; * *p* < 0.05). (**d**) The numbers of mast cells on day 50 were significantly lower in the alginate gel encapsulating dE1-k35/sLRP6E1E2-treated scar tissues (Group V) compared with other groups (* *p* < 0.05).

To examine inflammatory cell infiltration within the scar tissues, the number of inflammatory cells was calculated from four serial H&E-stained tissue sections (Figure 6a). On day 5 of the postoperative period, the mean numbers of inflammatory cells within the scar tissue were 9.2 ± 2.39, 9.25 ± 2.63, 5.80 ± 1.79, 7.75 ± 1.71, and 2.20 ± 1.30 in each group, respectively. On day 50 of the postoperative period, these were 9 ± 2.6, 8.5 ± 1.41, 8 ± 1.01, 6.28 ± 0.84, and 3.35 ± 0.30 in each group, respectively. The numbers of inflammatory cells on days 5 and 50 were significantly lower in the alginate gel encapsulating dE1-k35/sLRP6E1E2-treated scar tissues (Group V) (* *p* < 0.05; Figure 6b,c).

Mast cells are reported to be involved in the proliferation and contraction of fibroblasts, and the synthesis of ECM. They also play a key role in scar formation. On day 50 of the postoperative period, the mean numbers of mast cells were 10.17 ± 0.6, 8.97 ± 0.39, 8.53 ± 0.52, 7.03 ± 0.38, and 2.51 ± 0.25 in each group, respectively. A statistically significant decrease was seen in the mean number of mast cells in the alginate gel encapsulating dE1-k35/sLRP6E1E2-treated scar tissues (Group V) in comparison to other groups (* *p* < 0.05; Figure 6d).

## 3. Discussion

Keloids are benign fibroproliferative scars that invade surrounding normal skin beyond the original wound boundaries and keep growing slowly like a benign skin tumor [4]. The incidence has been reported at about 16% [11], and they are highly associated with dark, pigmented skin [12]. Unlike hypertrophic scars, they rarely regress spontaneously, resulting in both aesthetic problems and pain and functional disability. 

Various mechanisms have been suggested to explain keloid pathogenesis: altered growth factor regulation, immune dysfunction, aberrant collagen turnover, sebum or sebocytes as self-antigens, altered mechanics, and altered apoptotic signaling in keloid fibroblasts [1,13]. Although a consistent theory is still lacking, TGF-β1 is known as a potent fibrogenic growth factor that plays a primary role in keloid pathophysiology [2,14,15]. Thus, targeting TGF-β1 with pharmacologic interventions represents an attractive therapeutic option.

Skin injury involves the response of the Wnt/β-catenin pathway, with a critical role in epidermal stem cell maintenance, hair follicle development, regeneration, and fibroblast-mediated scarring [16]. Activation of the Wnt pathway plays a profibrotic role in cutaneous wound healing. Wnt/β-catenin signaling can upregulate TGF-β expression [17,18], and TGF-β1 can promote β-catenin signaling [19,20,21]. Furthermore, TGF-β stimulates canonical Wnt signaling by reducing the expression of the Wnt antagonist Dickkopf protein-1 [22]. Prolonged activation of Wnt/β-catenin signaling has been observed in human hyperplastic wounds [23]. Therefore, inhibition of the canonical Wnt pathway might be an effective approach to target TGF-β signaling in fibrotic diseases such as keloids or hypertrophic scars. 

We previously generated a novel soluble decoy Wnt receptor, sLRP6E1E2, which is composed of the low-density lipoprotein receptor-related protein 6 (LRP6) E1 and E2 regions, for functional interaction with Wnt and sLRP6E1E2-expressing replication-incompetent adenovirus (dE1-k35/sLRP6E1E2). As LRP6 plays a key role in the activation of the canonical Wnt signaling pathway, these replication-defective Ad-based vectors engineered with the soluble decoy Wnt receptor sLRP6E1E2 induce an anti-fibrotic effect by inhibiting Wnt and TGF-β signaling. The effect was observed in vitro with human dermal fibroblasts and keloid spheroids in a previous study [6]. 

Using a pig scar model, we confirmed its therapeutic effect in vivo. Macroscopically, this approach helps to decrease scar size and minimize discoloration. Microscopically, we observed that the collagen structure of scar tissue became similar to normal tissue after treatment. Expression of structural proteins was decreased in the treatment group. As with in vitro results, sLRP6E1E2-expressing Ad downregulated TGF-β1 and upregulated TGF-β3 mRNA expression in vivo. In addition, the expression of molecules that associated scar remodeling also convinced us of the anti-fibrotic effect of sLRP6E1E2-expressing Ad. The expression level of MMP-1, which involves the breakdown of extracellular matrix and tissue remodeling, breaking down the interstitial collagens, was increased in the Ad-treated group. In contrast, TIMP-1, which has a role in extracellular matrix remodeling by the inhibitory activity of MMP-1 [24,25], showed a decreased expression level as well as α-SMA expression. The mast cells, known to be involved in the proliferation and contraction of fibroblasts, and the synthesis of ECM, which play a key role in scar formation, are also observed in fewer numbers in the virus-treated group. The numbers of inflammatory cells were lower in the sLRP6E1E2-expressing Ad-treated group. Given that the keloid is the result of chronic inflammation of the reticula dermis [26], this result is also expected to provide a positive basis for treating problem scarring in the future. 

Although adenovirus is an attractive gene delivery vector with the advantages of high gene-transfer ability, easy production/amplification, and low risk of insertional mutagenesis, its major drawback is the rapid elimination of transduced cells [12]. As in vivo applications require long-term transduction, we introduced an Ad/alginate gel depot system. Previous studies indicated that the biological activity of Ad loaded in alginate gel is prolonged compared with that of naked Ad over an extended period of time [7]. In addition, encapsulation of viral vectors may enhance the therapeutic effect by masking viruses from clearance [9]. In this in vivo study, we confirm that alginate gel-encapsulated decoy Wnt expressing Ad is more effective in scar management compared to naked Ad in a pig model. The alginate gel reservoir releases Ad in a sustained manner and maintains the biological activity of Ad longer. Alginate gel has also been shown to limit the Ad mobility by entrapping Ad within its scaffold, further minimizing the spread of the replication-incompetent Ad outside the scar tissue. A microenvironment surrounded with alginate gel also protects the virus from clearance by the innate immune system.

## 4. Materials and Methods

### 4.1. Preparation of Ad and Alginate Gel

A replication-incompetent Ad expressing the Wnt decoy receptor relaxin (dE1-k35/sLRP6E1E2) and control Ad (dE1-k35/LacZ) [27] were used in this study. The propagation, purification, and titration of Ad were performed as previously described [28,29]. An alginate (alginic acid sodium salt, Sigma-Aldrich Co, St. Louis, MO, USA) solution of 5 wt % was prepared with 0.5 g alginate and 0.09 g NaCl (Sigma) in 10 mL phosphate-buffered saline (PBS). The alginate solution was stirred for 24 h at room temperature and gelated in 50 mM CaCl_2_ (Sigma) [7].

### 4.2. Biological Activity of Encapsulated Ad

To compare the biological activity of Ad in different forms, cellular transduction efficiency of naked Ad, Ad released from the gel, and Ad trapped in gel were measured by GFP expression. Ad (1 × 10^10^ viral particle of dE1/GFP) was encapsulated in 5% alginate gel and incubated with Dulbecco’s modified Eagle’s medium at 37 °C. The media containing released Ad (2 mL) was collected at days 1, 3, 5, 7, 9, 11, and 13. The remaining Ad/alginate gel was dissolved with gel dissolution solution and then 2 mL of cell culture media was added. SK-Hep 1 cells were then transduced with naked Ad, released Ad from the gel, and gel-encapsulated Ad. At 72 h post-transduction, GFP-expressing cells were identified by fluorescence microscopy (Olympus BX51; Olympus Optical, Tokyo, Japan). 

### 4.3. Scar Formation

A female Yorkshire pigs (4 months of age, 40 kg) were used for the scar model. This animal study was approved by an the Animal Care and Experiment Committee of Yonsei University (2013-0273, 8 October 2013). All animal experimental procedures were performed in accordance with the relevant guidelines and regulations of the Department of Laboratory Animal Resources, Yonsei Biomedical Research Institute, Yonsei University College of Medicine. Anesthesia was induced in each pig via intramuscular injection of Zoletil^®^ (5 mg/kg, Virbac, Carros, France) and Rompun^®^ (2 mg/kg, Bayer, Seoul, Korea) and maintained through inhalation of isoflurane (IsoFlo^®^, Abbott Laboratories, North Chicago, Illinois, USA). After complete hair removal, 36 full-thickness skin defects (3 × 3 cm^2^) were created symmetrically on the back of each Yorkshire pig, with 18 wounds on each side of the midline. The wound was spaced at least 3 cm apart and reached the depth of the muscle fascia to mimic human scar formation. Intravenous antibiotics were administered, and TegaDerm^®^ (3M, St Paul, MN, USA) was used as wound dressing for 5 days. Scar tissues, which were distinct in appearance compared to the peripheral normal tissues, were formed 50 days after creating the initial skin defects.

### 4.4. Injection of Ad

Wound epithelialization and scar formation occurred on postoperative day 50. Scars were divided into five treatment groups: (I), PBS (control, *n* = 7); (II), alginate gel (gel, *n* = 7); (III), alginate gel encapsulating dE1-k35/LacZ (gel+dE1-k35/LacZ, capsulated control, *n* = 7); IV, naked dE1-k35/sLRP6E1E2 (naked LRP6, *n* = 7); and V, alginate gel encapsulating naked dE1-k35/sLRP6E1E2 (gel+dE1-k35/sLRP6E1E2, capsulated LRP6, *n* = 8) (5 × 10^7^ PFU). Under anesthesia, viruses were injected with a 27-gauge needle with a 1-mL syringe, and Ad/alginate gel mixtures were directly injected into the intradermal layers of the scar regions. 

### 4.5. Therapeutic Evaluation of Scar Size and Erythema and Melanin Indices

The surface areas of the scars were imaged with a digital camera. The images were measured with a ruler and compared to a 1-cm^2^ standard, and measurements were assessed with ImageJ software (National Institutes of Health, Bethesda, MD, USA). Scar color was quantitatively analyzed with a spectrophotometer (CM-700D; Konica Minolta, Inc., Tokyo, Japan). The color of each scar was examined with erythema (the degree of scar redness) and melanin (the degree of scar darkness) indices [30]. Each value was measured at least three times, and the mean was calculated. All of the evaluations were performed every 10 days until the 50th day after virus injection. We normalized the values of alginate gel (gel), alginate gel encapsulating dE1-k35/LacZ (capsulated control), naked dE1-k35/sLRP6E1E2 (naked LRP6), and alginate gel encapsulating dE1-k35/sLRP6E1E2 (capsulated LRP6)-treated scars to that of PBS-treated scars.

### 4.6. Histology and Immunohistochemistry

Representative sections collected 5 and 50 days after experiments were stained with Picrosirius red (Sigma-Aldrich Co.) for evaluation of collagen-fiber arrangement with optical microscopy (BX51, Olympus, Tokyo, Japan) at ×200 magnification. Immunohistochemistry was performed by the same individual who was blinded to the experimental group and the tissue sections for analysis were obtained from the center areas of scar tissue. Scar tissue sections were incubated at 4 °C overnight with one of the following primary antibodies: mouse anti-collagen type-I (ab6308; Abcam, Ltd., Cambridge, UK), mouse anti-elastin (E4013; Sigma-Aldrich Co. St. Louis, MO, USA), and mouse anti-fibronectin (sc-52331; Santa Cruz Biotechnology, Santa Cruz, USA). Then, tissues were incubated at room temperature for 20 min with the DAKO Envision™ Kit (DAKO, Glostrup, Denmark) as a secondary antibody. Diaminobenzidine/hydrogen peroxidase (DAKO) was used as the chromogen substrate. All sections were counterstained with Meyer’s hematoxylin. Expression levels of type I collagen, elastin, and fibronectin were semi-quantitatively analyzed with MetaMorph^®^ image analysis software (Molecular Devices, Sunnyvale, CA, USA). Values are calculated as the mean optical density of six different digital images. 

### 4.7. Real-Time Reverse Transcriptase-Polymerase Chain Reaction (RT-PCR) 

At 50 days after Ad injection, total RNA was prepared from tissue with TRIzol^®^ reagent (Invitrogen, Carlsbad, CA, USA), and complementary DNA was prepared from 500 ng total RNA by Oligo dT (Bioneer Corp., Alameda, CA, USA) and a cDNA synthesis kit (Bioneer Corp.) under the following conditions: 42 °C for 60 min, 94 °C for 5 min. Real-time PCR was performed with a StepOnePlus™ Real-Time PCR System (Thermo Fisher Scientific, Waltham, MA, USA), following a Taqman^®^ Real-time PCR assay (Thermo Fisher Scientific; assay ID: Ss03382325_u1 (TGF-β1), Ss03394349_g1 (TGF-β3), Ss04245657_g1 (matrix metalloproteinase-1, MMP-1), Ss03381944_u1 (tissue inhibitor of metalloproteinase 1, TIMP-1), and Ss04245588_m1 (α-smooth muscle actin, α-SMA)). Target mRNA expression levels were normalized to that of β-actin (Thermo Fisher Scientific; assay ID: Ss03376563_uH) levels, and relative quantization was expressed as fold-induction compared with control conditions in each tissue. PCR was performed three times for each mRNA in each tissue, and the average value was calculated.

### 4.8. Statistics

Results are expressed as the mean ± standard deviation (SD). Data were analyzed with repeated-measures one-way analysis of variance (ANOVA). Two sets of independent sample data were compared using paired *t*-tests; *p* < 0.05 was considered statistically significant.

## 5. Conclusions

Decoy Wnt receptor (sLRP6E1E2)-expressing adenovirus treatment improved scar quality in a pig model. Loading this construct in alginate gel allows sustained virus release into local tissues and prolongs Ad activity, thus maintaining its therapeutic effect longer in vivo. These advantages of the Ad/alginate system could markedly augment the scar remodeling effects of sLRPE1E2 on scar tissue by steadily providing a high concentration of Ad with minimal toxicity.

## Abbreviations

Ad	Adenoviral vector
ECM	Extracellular matrix
TGF-β	Transforming growth factor-β
PBS	Phosphate-buffered saline
GFP	Green Fluorescent Protein
